# Differentiating clinically important interstitial lung abnormalities in lung cancer screening

**DOI:** 10.1136/bmjresp-2025-003298

**Published:** 2025-09-10

**Authors:** Brintha Selvarajah, Amyn Bhamani, Mehran Azimbagirad, Burcu Ozaltin, Ryoko Egashira, Daisuke Yamuda, John McCabe, Nicola Smallcombe, Priyam Verghese, Ruth Prendecki, Andrew Creamer, Jennifer L Dickson, Carolyn Horst, Sophie Tisi, Helen Hall, Chuen R Khaw, Monica L Mullin, Kylie Gyertson, Anne-Marie Hacker, Laura Farrelly, Anand Devaraj, Arjun Nair, Mariia Yuneva, Neal Navani, Daniel C Alexander, Rachel Clare Chambers, Joanna Porter, Allan Hackshaw, Gisli Jenkins, The SUMMIT Consortium, Sam M Janes, Sam M Janes, Joseph Jacob

**Affiliations:** 1 Centre for Inflammation and Tissue Repair, UCL Respiratory, University College London, London, UK; 2 Oncogenes and Tumour Metabolism Lab, The Francis Crick Institute, London, UK; 3 University College London Hospitals NHS Foundation Trust, London, UK; 4 Lungs for Living Research Centre, UCL Respiratory, University College London, London, UK; 5 Satsuma Lab, Centre for Medical Image Computing, University College London, London, UK; 6 Vocational School of Health Services, Kahramanmaras Sutcu Imam University, Kahramanmaras, Turkey; 7 Department of Radiology, Faculty of Medicine, Saga University, Saga, Japan; 8 Royal London Hospital, Barts Health NHS Trust, London, UK; 9 The University of British Columbia, Vancouver, British Columbia, Canada; 10 Cancer Research UK and UCL Cancer Trials Centre, University College London, London, UK; 11 Royal Brompton and Harefield Hospitals, London, UK; 12 National Heart and Lung Institute, Imperial College London, London, UK; 13 UCL Centre for Medical Image Computing, Department of Computer Science, University College London, London, UK

**Keywords:** Idiopathic Pulmonary Fibrosis, Interstitial Fibrosis, Imaging/CT MRI etc

## Abstract

**Background:**

Interstitial lung abnormalities (ILAs) are common incidental findings in lung cancer screening (LCS). However, challenges remain in identifying clinically relevant ILAs as highlighted in a joint statement by a European multidisciplinary task force led by the European Respiratory Society (ERS). To address these challenges, we analysed ILAs identified in one of Europe’s largest LCS studies.

**Methods:**

Of 11 635 LCS individuals, 417 screen-detected ILAs were evaluated using a new visual classification system focused on traction bronchiolectasis: non-fibrotic ILA (no traction bronchiolectasis), fibrotic ILA (traction bronchiolectasis in ≤2 lobes); undiagnosed interstitial lung disease (traction bronchiolectasis in >2 lobes). Observer agreement was compared with Fleischner Society ILA classification using Cohen’s Kappa. An age, sex and smoking history-matched control group allowed the examination of associations between baseline ILA/UILD and comorbidities, forced vital capacity (FVC), hospitalisations (Student’s t-tests) and mortality (univariable and multivariable Cox proportional hazards models).

**Findings:**

Our visual ILA classification showed superior interobserver agreement (K=0.76) versus the Fleischner ILA classification (K=0.64). ILA/UILD subjects had more prevalent comorbidities, increasing (vs controls) approximately 10 years prior to ILA/UILD diagnosis. Compared with controls, mortality rates were 6-fold higher for UILD participants and 3-fold higher for fibrotic and non-fibrotic ILA subtypes. On multivariable Cox regression analysis, ILA/UILD presence (HR=4.90, 95% CI =2.36 to 10.10, p<0.001) showed stronger independent associations with mortality than baseline FVC (HR=0.98, 95% CI =0.96 to 1.00, p=0.04).

**Conclusion:**

We demonstrate a new reproducible classification of clinically important ILA/UILDs in LCS populations. We highlight that FVC shows limited associations with mortality in ILA/UILD subjects. Increased multiorgan comorbidity in ILA/UILD subjects highlights a need for comprehensive early multisystem evaluation.

**WHAT IS ALREADY KNOWN ON THIS TOPIC**

**WHAT THIS STUDY ADDS**

- Given previously reported challenges in estimating ILA presence, we demonstrate a new reproducible radiological classification to characterise clinically important ILAs and ILD in SUMMIT, one of the largest lung cancer screening studies (LCS) in the world.

**HOW THIS STUDY MIGHT AFFECT RESEARCH, PRACTICE OR POLICY**

- The findings of this study present a reproducible method to identify clinically important ILAs in LCS populations and have important implications regarding the management of ILAs to improve comorbid burden and mortality.

## Introduction

 Low-dose CT (LDCT) lung cancer screening (LCS) programmes allow for the early detection and treatment of lung cancer.^1^ Subjects invited for LCS are also at risk for the development of lung fibrosis.^2^ Interstitial lung abnormalities (ILAs) are incidental parenchymal CT abnormalities that commonly occur in the ageing population, with a reported prevalence of between 3% and 10% in LCS cohorts.^3^ ILAs are associated with increased all-cause mortality and may represent an early stage of lung fibrosis.^3 4^ With nearly one million participants predicted to undergo LDCT in England annually by 2030 as part of a national LCS programme, there is a pressing clinical need to distinguish clinically relevant ILAs.

A recent multidisciplinary European statement on the management of incidental findings from CT LCS, coordinated by the European Respiratory Society (ERS) in collaboration with the European Society of Thoracic Surgeons (ESTS), European Society for Radiation Oncology (ESTRO), European Society of Radiology (ESR), European Society of Thoracic Imaging (ESTI), and the European Federation of Organisations for Medical Physics (EFOMP), highlighted key research questions related to screen-detected ILAs. ^3^ These included the need to find optimal ways to differentiate ILAs from interstitial lung diseases (ILD) and how best to characterise ILAs. We aimed to address these challenges by analysing ILAs identified visually in the SUMMIT Study, one of the largest LCS studies in the world.^5^ We examined how ILA subtypes associate with symptom progression, hospitalisation and mortality.

## Methods

### Study cohort

SUMMIT is a prospective, longitudinal cohort study aiming to assess the implementation of LDCT screening for lung cancer in a high-risk population in London (NCT03934866). The pre-SARS-CoV-2 (COVID-19) pandemic recruitment period (08 April 2019 to 19 March 2020) invited 55–77 year-olds, who smoked within the past 20 years and had a predefined cancer risk (online supplemental appendix) for clinical evaluation, symptom questionnaires and CT.^5 6^ The baseline observations of the SUMMIT screening study have recently been reported.^7^

Chronic productive cough, breathlessness (Modified Medical Research Council (mMRC) grades) and antibiotic and/or steroid use in the preceding 12 months were assessed. Participants were asked, as part of the SUMMIT screening questionnaire, whether they had a job working with asbestos, coal dust, wood dust or other minerals, rubber or metal dusts without using protective equipment. Following baseline CT, participants were invited for lung health check appointments at 12 months and 24 months and repeat CT at 24 months. Spirometry (unless clinically contraindicated) was comprehensively performed before but only sporadically after the pandemic (March 2020), precluding longitudinal spirometric analysis. Subjects without baseline spirometry and individuals with: (a) known history of ILD and (b) lung cancer evident on initial CT were also excluded from this analysis (online supplemental figure 1). Ethical approval for the SUMMIT Study and ongoing analyses was obtained from a National Health Service (NHS) research ethics committee (17/LO/2004) and the NHS Health Research Authority’s confidentiality advisory group (18/CAG/0054).

### ILA identification

Non-contrast inspiratory volumetric (0.625 mm slice thickness; General Electric Revolution scanners) CT images were reported contemporaneously by consultant thoracic radiologists. Initial screening reported ILA classification included: mild (<10% reticulation), moderate (>10% reticulation without fibrotic features) or severe (>10% reticulation with fibrotic features). All CT time points in ILA subjects were adjudicated by an independent specialist thoracic radiologist (JJ) to confirm ILA presence (online supplemental figure 1). Subjects where abnormalities resolved on subsequent CTs (infection, inflammation or suboptimal lung expansion on initial CT) were excluded from analysis (online supplemental figure 2). A control group of subjects without ILAs on two time point CTs (confirmed by JJ) was 1:1 matched with the ILA cohort using sex (exact match), age (±2 years) and smoking pack-year history (nearest pack-years).

Given previously reported challenges in estimating ILA presence using Fleischner Society ILA criteria (>5% in one zone) and ILA subtypes,^4 8 9^ a new ILA classification system which did not require assessment of lung volume percentages was examined. ILA was assigned for incidental findings of non-dependent CT parenchymal abnormalities. A focus of our scoring system was the delineation of traction bronchiolectasis as an early manifestation of fibrosis. This contrasted with the Fleischner Society criteria which focus on traction bronchiectasis, honeycombing and architectural distortion which represent features of advanced fibrosis.

**Figure 1:**
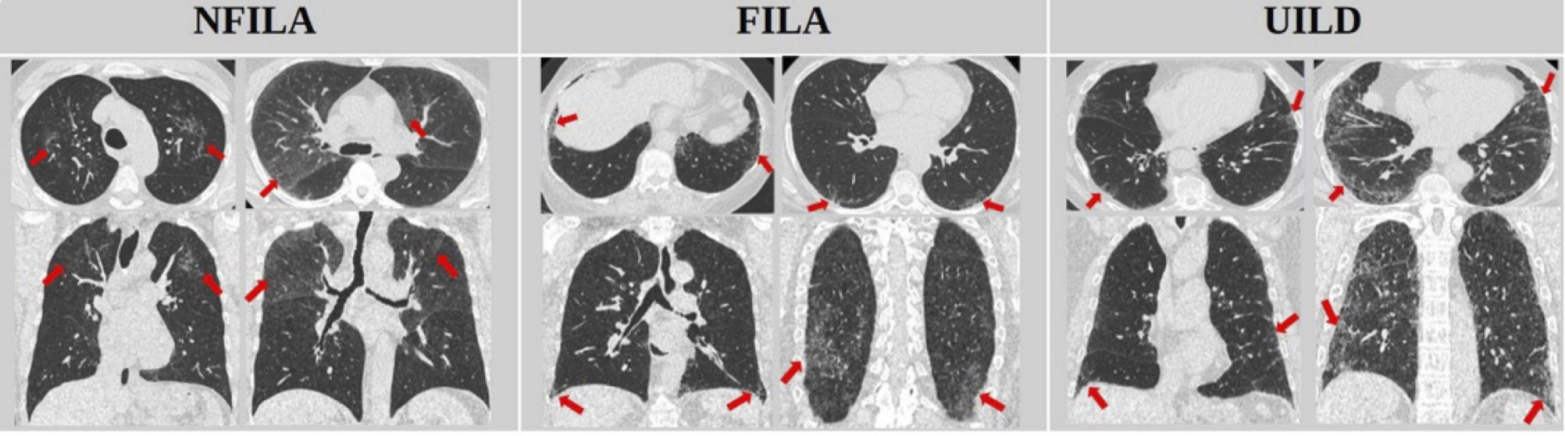
Axial and coronal CT examples of non-fibrotic interstitial lung abnormality (NFILA), fibrotic interstitial lung abnormality (FILA) and undiagnosed interstitial lung disease (UILD) identified in the SUMMIT cohort. NFILA required the presence of non-dependent ground glass opacities and/or reticulation with no associated traction bronchiolectasis evident in any lobes. FILA required traction bronchiolectasis (coexisting with reticulation with/ without non-dependent ground glass opacities) to be evident in a maximum of 2 lobes. UILD required traction bronchiolectasis (coexisting with reticulation with/ without non-dependent ground glass opacities) to be evident in more than two lobes.

Three ILA/UILD subtypes were defined: (a) non-fibrotic ILA (NFILA): non-dependent ground glass opacities (GGO) and/or reticulation, without traction bronchiolectasis in any lobes; (b) fibrotic ILA (FILA): traction bronchiolectasis (coexisting with reticulation with/without GGO) evident in a maximum of 2 lobes; (c) undiagnosed ILD (UILD): traction bronchiolectasis (coexisting with reticulation with/without GGO) evident in more than two lobes (figure 1). Interobserver agreement for Fleischner and our CT criteria was tested by two pairs of subspecialist thoracic radiologists in two ways: (a) 50% of the control and ILA subjects were assessed (RE: 23-year experience; JJ 16-year experience); (b) ILA subtype agreement was assessed (DY: 14-year experience; JJ 16-year experience).

### Comorbidities and clinical outcomes

Prevalent (present in general practice records before study consent date) comorbidities were documented. Information on emergency, respiratory and all-cause hospital admissions was obtained from the NHS Digital Hospital Episode Statistics database. Cancer diagnoses and mortality outcomes were obtained from the National Cancer Registration and Analysis Service (NCRAS) Rapid Cancer Registration. Total CT coronary calcification scores were calculated by summation of reporting radiologist-assigned CT coronary calcification scores in the circumflex, left anterior descending and right coronary arteries (none=0, minimum=1, mild=2, moderate=3, severe=4). Residential postcodes obtained from primary care records were converted into 2019 English Index of Multiple Deprivation (IMD) ranks to provide a measure of socioeconomic deprivation.

### Statistical analysis

Data are presented as patient proportions (percentages), means (with SD) or medians (with range of values), as appropriate. Differences in categorical variables were assessed using the χ^2^ test. Differences in medians of continuous variables were assessed using the two-sided Mann-Whitney U test. Differences in means of continuous variables were assessed using the two-sided Student’s t-test. In three-group comparisons, a Kruskal-Wallis rank sum test evaluated differences in medians and a one-way analysis of variance (ANOVA) evaluated differences in means. Interobserver variation for visual CT scores was assessed using Cohen’s Kappa (binary variables) and weighted Kappa (ordinal variables).

Median survival times and their respective 95% CIs were determined using Kaplan-Meier curves with group differences assessed using the log rank test. Follow-up time represented the interval between baseline CT and death or the last available NCRAS download date (18 September 2023). Univariable and multivariable Cox proportional hazards models assessed time to death for either the presence of ILA/UILD or an ordinal classification of no-ILA, NFILA, FILA and UILD. Adjusted covariates in all multivariable Cox models included: patient age, sex, smoking pack-years and percent predicted forced vital capacity (FVC) at baseline CT. Multivariable Cox regression models separately examined the effect of deprivation, cumulative prevalent comorbidities (none, one, two, three or more than three comorbidities), body mass index (BMI) and coronary calcium scores on survival. Schoenfeld residuals checked the proportional hazards assumption, martingale residuals assessed nonlinearity and deviance residuals (symmetric transformation of the martingale residuals) examined influential observations. A p value<0.05 was considered significant across all analyses. Data analysis/preparation was performed in R V.3.6.9, using R survival analysis packages: ‘survival’, ‘ggplot2’, ‘survminer’ and ‘forestplot’.

### Patient and public involvement

The protocol, study design and supporting documents for this study underwent review by a participant and public involvement group on several occasions. This included invitation materials, participant information sheet, consent form and results letters, which were all reviewed by patient and public representatives for their readability and acceptability. This was an ongoing process and several of the members of this group continued to be involved by being included on the study steering committee.

## Results

### ILA categorisation

Of 555/11 635 (4·8%) individuals with baseline CT screening ILAs diagnosed by a reporting radiologist (online supplemental figure 1), 417/555 (75.1%) ILA CTs were examined after specialist re-review and exclusion of subjects with known ILDs, missing data and study withdrawal. 417 matched subjects without ILAs formed the control population (online supplemental figure 1).

Interobserver agreement between radiologists (JJ and RE) using the Fleischner Society ILA criteria was K=0·64. Using our criteria, agreement for ILA/UILD presence (K=0·76) and ILA subtypes (no ILA, NFILA, FILA and UILD: K=0·84) was better. Interobserver agreement for ILA/UILD subtypes (JJ and DY) using the Fleischner Society criteria (NFILA K=0·42; FILA K=0.26, UILD K=0.51) was worse than when using our criteria (NFILA K=0·86; FILA K=0.82 UILD K=0.94) (online supplemental table 1). While our criteria formed the cardinal classification criteria for all further analyses, results using the Fleischner Society criteria are included in (online supplemental tables 1, 5-9).

### Baseline ILA/UILD demographics

Study participants were predominantly men (68·8%), with a mean age of 68·0 years, a 45-pack-year smoking history (table 1) and a high current smoking proportion (46%). No difference in self-reported chronic obstructive pulmonary disease(COPD) diagnoses was found between ILA/UILD and non-ILA subjects (p=0·14). Of the 417 subjects identified as having ILA/UILDs: 117 (28.1%) participants were classified as NFILA, 138 participants (33·1%) as FILA and 162 (38·8%) as UILD (table 2).

**Table 1:** Demographic data for subjects with and without interstitial lung abnormalities/disease (ILA/UILD) identified on CT imaging

N (%)	ILA/UILD	No ILA	P value
Demographics	417	417	
Age (years)	68.03	68.03	
Female	130 (31.2)	130 (31.2)	
Male	287 (68.8)	287 (68.8)	
Ethnicity			
White	345 (82.7)	343 (82.3)	
Black	25 (6.0)	14 (3.4)	
Asian	32 (7.7)	35 (8.4)	
Mixed	5 (1.2)	11 (2.6)	
Other	10 (2.4)	14 (3.4)	
Unknown	–	–	
Deprivation Index Quintile (mean, SD)	2.25 (1.2)	2.44 (1.2)	0.019
BMI (mean, SD)	28.60 (5.1)	27.24 (5.0)	<0.001
Current smoker (%)	191 (45.8)	197 (47.2)	0.677
Smoking history (pack years) (mean) (SD)	44.97 (20.2)	44.95 (20.3)	0.991
Systolic blood pressure (mean, SD)	135.87 (17.0)	134.77 (16.4)	0.345
Total CT calcium scoring (mean, SD)	5.27 (3.7)	4.11 (3.7)	<0.001
History of occupational exposure (%)	99 (23.7)	69 (16.5)	<0.01
Lung function			
FEV1/FVC ratio (mean, SD)	0.70 (0.1)	0.67 (0.1)	<0.001
FEV1 (L)	2.21 (0.6)	2.14 (0.7)	0.129
FEV1 (% predicted)	78.70 (17.3)	76.71 (2)	0.145
FVC (L)	3.17 (0.8)	3.17 (0.9)	0.989
FVC (% predicted)	87.28 (15.8)	87.39 (18.1)	0.925
No. with FVC <80% (%)	129 (30.9)	121 (29.0)	0.545
Symptoms			
Cough present (%)	162 (38.8)	141 (33.8)	0.131
Antibiotic and/or steroid courses in last 12 months (mean, SD)	0.42 (1.0)	0.26 (0.7)	0.006
Breathlessness			
mMRC score (mean, SD)	1.06 (1.0)	0.97 (1.0)	0.170
No. with mMRC score ≥2(%)	97 (23.3)	83 (19.9)	0.241
COPD diagnosis before initial CT (%)	127 (30.5)	108 (25.9)	0.144
Hospital admissions			
Total yearly hospital admissions (mean, SD)	0.17 (0.5)	0.16 (0.4)	0.585
Total yearly respiratory admissions (mean, SD)	0.03 (0.1)	0.03 (0.1)	0.760
Mortality			
No. died (%)	39 (9.4)	9 (2.2)	<0.001
Deaths secondary to:			
COVID-19	7	2	
Respiratory disease	4	2	
Lung cancer	5	0	
Other cancers	4	1	
Cardiac disease	9	1	
Cerebrovascular disease	6	0	
Other	3	0	
Unknown	1	3	
Time to death (years) (mean, SD)	2.11 (1.1)	1.95 (1.1)	0.702
Overall follow-up time (years) (mean, SD)	3.71 (0.7)	3.86 (0.4)	<0.001

BMI, body mass index; FEV1, forced expiratory volume in 1 second; FVC, forced vital capacity; mMRC, Modified Medical Research Council.

**Table 2:** Demographic data for subjects with interstitial lung abnormalities subtypes identified on CT imaging

N (%)	NFILA	FILA	UILD
Demographic	117 (28.1)	138 (33.1)	162 (38.8)
Age (years)	66.36	68.59	68.76
Female	41 (35.0)	50 (36.2)	39 (24.1)
Male	76 (65.0)	88 (63.8)	123 (75.9)
Ethnicity			
White	98 (83.8)	115 (83.3)	132 (81.5)
Black	10 (8.5)	6 (4.4)	9 (5.6)
Asian	7 (6.0)	8 (5.8)	17 (10.5)
Mixed	0	4 (2.9)	1 (0.6)
Other	2 (1.7)	5 (3.6)	3 (1.8)
Unknown			
Deprivation Index Quintile (mean, SD)	2.39 (1.2)	2.26 (1.2)	2.13 (1.2)
Systolic blood pressure (mean, SD)	136.1 (16.3)	134.7 (16.0)	136.7 (18.2)
BMI (mean, SD)	28.6 (5.0)	29.3 (5.1)	28.0 (5.1)
Smoking history (mean pack years)	43.0 (20.3)	45.2 (20.2)	46.2 (20.4)
Current smoker (%)	57 (48.7)	61 (44.2)	73 (45.0)
Total CT coronary calcification (mean, SD)	4.58 (3.9)	5.53 (3.8)	5.56 (3.6)
History of occupational exposure (%)	30 (25.6)	29 (21.0)	40 (24.7)
Lung function			
FEV1/FVC ratio (mean, SD)	0.69 (0.1)	0.70 (0.1)	0.71 (0.1)
FEV1 (L)(mean, SD)	2.23 (0.7)	2.19 (0.6)	2.20 (0.6)
FEV1 (% predicted)(mean, SD)	78.72 (17.9)	79.50 (17.3)	78.01 (16.9)
FVC (L) (mean, SD)	3.26 (0.9)	3.15 (0.7)	3.12 (0.8)
Mean FVC (% predicted) (mean, SD)	88.69 (17.2)	88.54 (13.9)	85.19 (16.1)
No. with FVC<80% (%)	34 (29.1)	32 (23.2)	64 (39.5)
Hospital admissions			
Total yearly hospital admissions (mean, SD)	0.17 (0.5)	0.2 (0.6)	0.16 (0.4)
Total yearly respiratory admissions (mean, SD)	(0.0)	0.04 (0.1)	0.05 (0.2)
Symptoms			
Cough present (%)	46 (39.3)	52 (37.7)	64 (39.5)
Antibiotic and/or steroid courses in last 12 months (mean, SD)	0.44 (0.8)	0.33 (0.8)	0.49 (1.3)
Average mMRC score	1.04 (1.1)	1.05 (0.99)	1.11 (1.09)
No. with mMRC ≥2 (%)	29 (24.8)	30 (21.7)	38 (23.5)
Mortality			
No. died (%)	9 (7.8)	9 (6.5)	21 (13.0)
Deaths secondary to:			
COVID-19	2	-	5
Respiratory disease	1	2	1
Cardiovascular disease	3	3	3
Other	1	1	1
Lung cancer	-	1	4
Cerebrovascular disease	1	2	3
Other cancer	1	-	3
Unknown		-	1
Time to death (years) mean, SD	2.31 (1.3)	2.60 (0.7)	1.81 (1.2)
Overall follow-up time (years) (mean, SD)	3.77 (0.6)	3.79 (0.4)	3.60 (0.8)
No. of patient referred to ILD clinic from screening (%)	8 (6.8)	17 (12.3)	71 (43.8)

BMI, body mass index; FEV1, forced expiratory volume in 1 second; FILA, fibrotic interstitial lung abnormality; FVC, forced vital capacity; mMRC, Modified Medical Research Council; NFILA, non-fibrotic interstitial lung abnormality; UILD, undiagnosed interstitial lung disease.

**Figure 2:**
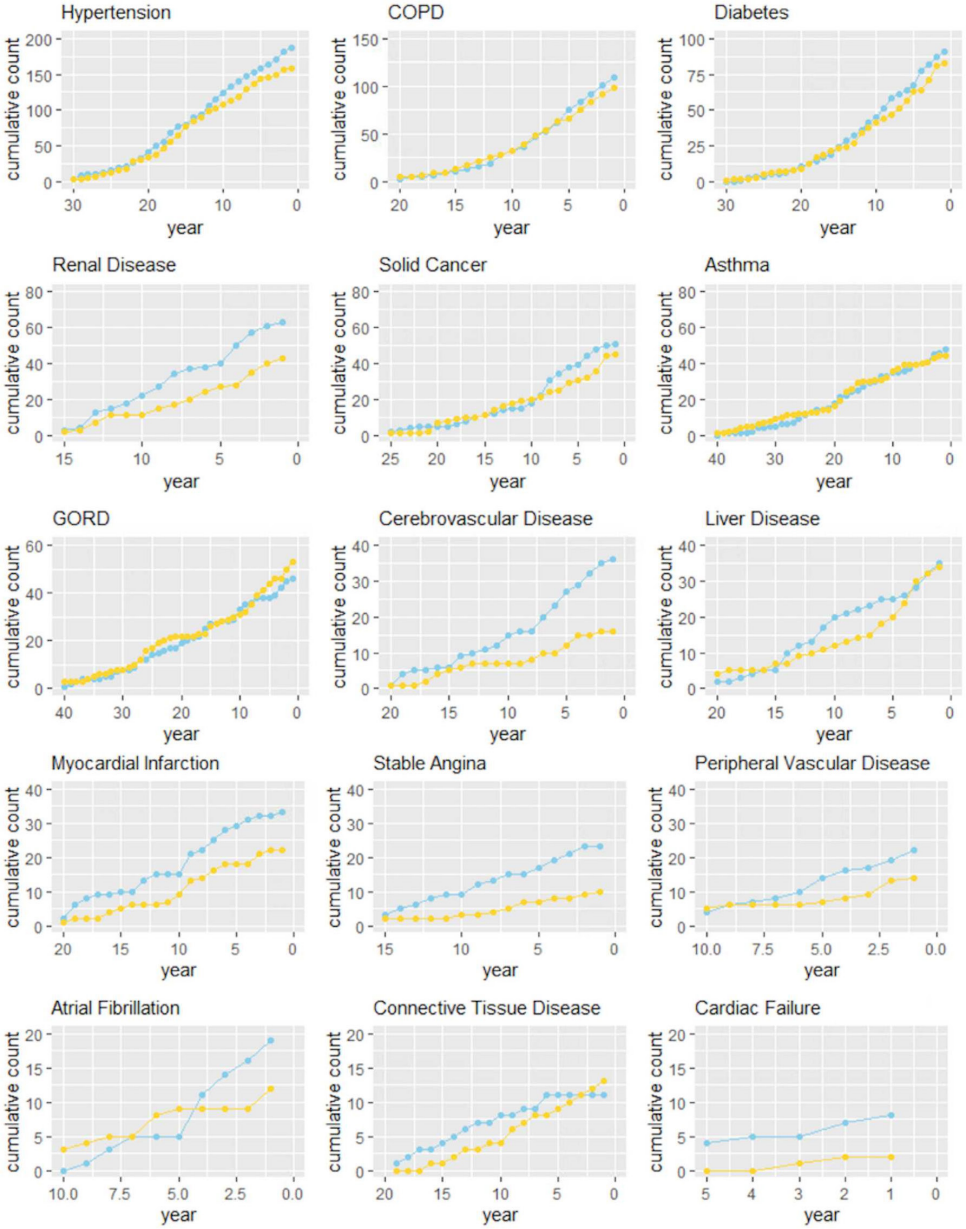
Curves showing cumulative incidence of disease comorbidities in the years prior to the initial lung cancer screening CT scan. Zero on the x-axis is the date of the initial CT scan. Separate curves are shown for subjects with (light blue) and without (yellow) interstitial lung abnormalities identified on CT. Dx, disease; COPD, chronic obstructive pulmonary disease; GORD, gastro-oesophageal reflux disease.

**Figure 3:**
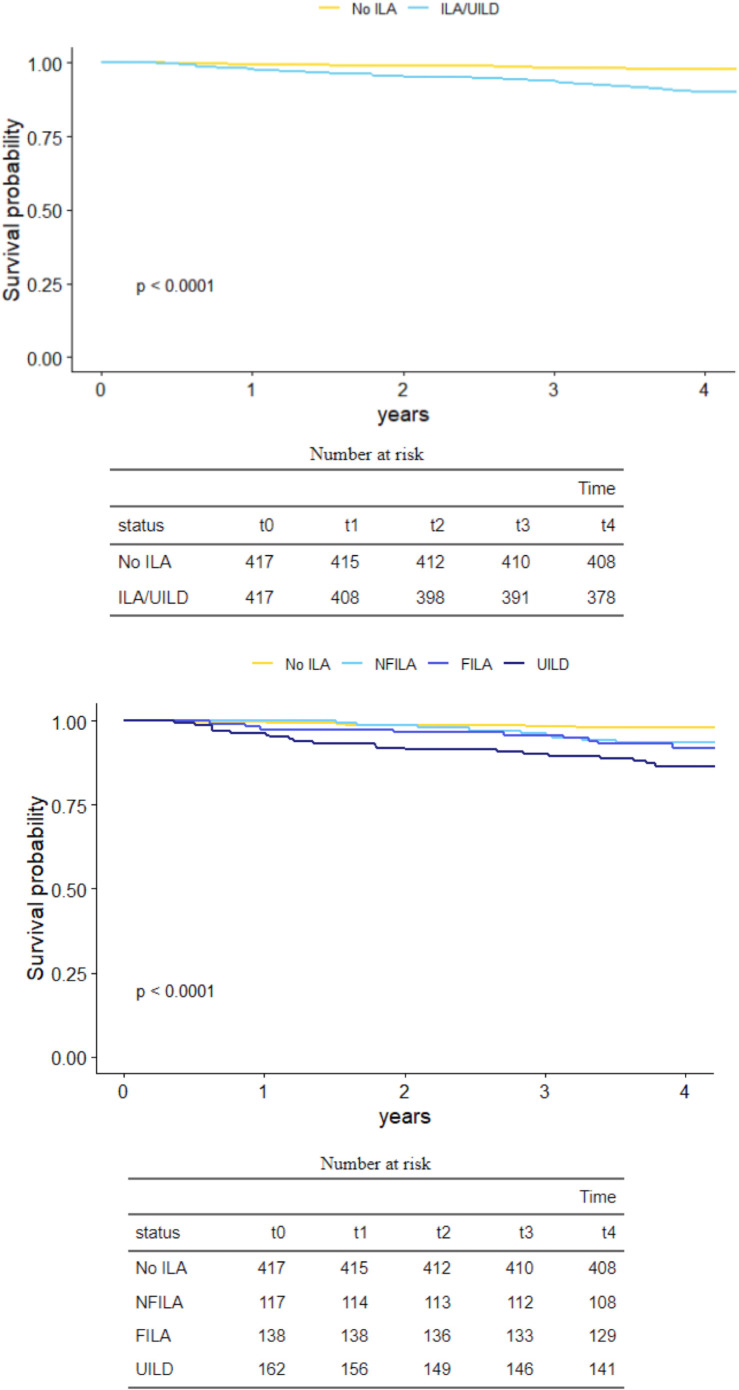
Kaplan-Meier survival curves for participants with and without interstitial lung abnormalities/disease (ILA/UILD) on CT (top) and for participants with and without subtypes of ILA/UILD on CT (bottom). FILA, fibrotic ILA; NFILA, non-fibrotic ILA; UILA, undiagnosed ILA.

Subjects with ILA/UILD showed greater deprivation (p=0·02), elevated BMI (p<0·001), increased self-reported occupational exposure (p<0.01), increased total coronary calcium scores (p<0·001), more prevalent comorbidities, in particular, cardiovascular, renal and cerebrovascular diseases (some evident 10 years prior to the first SUMMIT CT (figure 2, online supplemental table 2) and more restrictive spirometry (p<0·001), the latter driven largely by the UILD subgroup (tables1  2). There were no differences in mMRC scores or cough between ILA/UILD and non-ILAs or between ILA and UILD subgroups. ILA/UILD subjects reported more antibiotic or steroid use in the preceding 12 months (p=0·006), than non-ILA subjects particularly in the UILD group (tables1  2). Prevalent comorbidity frequency was similar across ILA/UILD subgroups (online supplemental table 3, figure 3).

### Clinical outcomes of ILA/UILD and control subjects

A greater proportion of participants with ILA/UILDs reported worsening mMRC scores over time (p=0·009) (online supplemental table 4), which was more pronounced in the UILD subgroup. The presence of cough did not increase in the ILA/UILD cohort over time; however, ILA/UILD subjects had increased overall hospital admissions (p=0·005) and respiratory hospital admissions (p=0·04) compared with non-ILA subjects, the latter most marked in the UILD subgroup (tables1  2).

Kaplan-Meier survival curves (figure 3) highlighted increased mortality in ILA/UILD participants (p<0·0001), with results maintained when COVID-19-related deaths were excluded from analyses (online supplemental figure 4). ILA/UILD deaths were predominantly coded as cardiac, respiratory, cerebrovascular disease, cancer and COVID-19 (table 1). NFILA and FILA subjects had a three-fold increased rate of mortality which increased to a six-fold increased mortality rate in UILD subjects compared with controls.

On multivariable Cox regression analysis, adjusted for age, sex, smoking status and percent predicted FVC at the time of the CT, ILA/UILD presence (HR=4·90, 95% CI= 2·36 to 10·10, p<0·001) was strongly associated with an increased risk of death, in contrast to FVC (HR=0·98, 95% CI =0.96 to 1·00, p=0·04). Results were maintained when cumulative prevalent comorbidities were considered in the multivariable model. When ILAs were considered using an ordinal scale (0=No ILA, 1=NFILA, 2=FILA, 3=UILD), worsening ILA/UILD severity associated with worse survival (HR=1·68, 95% CI=1·33–2·12, p<0·001). Results were maintained when either COVID-19-related deaths were excluded or when UILD subjects were excluded. Mortality associations using our scoring system were at worst equivalent to those using the Fleischner scoring system, but importantly identified more deaths in ILA/UILD subjects (online supplemental tables 5-9). Separate univariable and multivariable analyses of BMI, IMD ranks, obstructive spirometry (FEV1/FVC<0.7), occupational exposure history and CT coronary calcification scores similarly showed no significant associations with mortality.

## Discussion

Our study, evaluating one of the world’s largest LCS cohorts, examined a new reproducible method of classifying ILAs focused on traction bronchiolectasis, in order to address questions related to screen detected ILAs raised in a recent ERS/ESTS/ESTRO/ESR/ESTI/EFOMP statement.^3^ Our study identified four novel findings. First, our ILA classification showed improved observer agreement, identified more subjects with ILA/UILD who died during follow-up and demonstrated at least equivalent prognostic capabilities compared with the Flesichner Society ILA classification. Second, we distinguished two groups of ILA/UILD participants: UILD subjects who had a 6-fold increase in mortality and NFILA and FILA subjects with a 3-fold increase in mortality compared with subjects without ILAs. Importantly, mortality associations were not solely determined by UILD subjects as they remained significant for NFILA and FILA subjects when UILD subjects were excluded from analyses. Third, we demonstrated an increased prevalent comorbidity burden in ILA/UILD subjects compared with controls, which could be seen to occur ten years before the initial screening CT scan. Finally, we highlighted the limitations of FVC in distinguishing ILAs from controls and its limitations in identifying subjects with increased mortality risk in a heavy-smoking population.

The Fleischner Society threshold to classify ILAs (>5% non-dependent lung abnormalities in any zone) was acknowledged in the cardinal publication by the group as being arbitrary and imprecise. One key challenge when assessing lung volume is that even to expert observers, 5% of the lung can comprise different volumes of tissue. And this variability is only further complicated by the non-linear shrinkage of lung tissue that accompanies fibrotic remodelling. Our study confirmed that an ILA classification method that did not rely on volume assessments could be more reproducible than a method evaluating 5% volume thresholds.^4 8 9^ And by focusing on traction bronchiolectasis, which has been previously shown to be a prognostically important early sign of peripheral fibrosis,^10^ we demonstrated improved reproducibility for ILA/UILD subtype detection compared with the Fleischner criteria.

Our ILA criteria addressed a key point raised by the ERS/ESTS/ESTRO/ESR/ESTI/EFOMP statement, namely how best should ILA and ILD be consistently differentiated.^3^ The identification of ILD subjects defined a high-risk cohort with increased respiratory hospital admissions that should be referred to ILD services. Comparing our method of ILA/UILD classification to the Fleischner criteria, we identified more deaths in UILD, and the single extra death detected in the FILA/NFILA group using the Fleischner criteria had been classed as UILD by our criteria. Conversely, 10% of the deaths identified as ILA or UILD using our criteria were classified as non-ILA using the Fleischner criteria.

Compared with previous LCS studies, we report a higher prevalence of participants with FILAs and UILD. This is likely to relate to our utilisation of an early fibrotic marker, traction bronchiolectasis, which reclassified subjects from NFILA to FILA. Mortality rates between NFILA and FILA were similar, which is at first glance surprising. Even adjusting for cumulative prevalent comorbidities and smoking pack year history, the presence of NFILA/FILA is independently associated with increased mortality. However, with recent increased recognition that normal or minimally damaged areas on CT show fibrosis when viewed microscopically, it is probable that some foci of pure ground glass in subjects classed as NFILA did in fact contain microscopic fibrosis not visible on CT.^11 12^

For subjects with NFILA and FILA, the increased rate of multiorgan comorbidities (renal, cardiorespiratory, cerebrovascular disease) replicates observations on ILA comorbidity burden made in the AGES-Reykjavik, Framingham and SCAPIS studies^13 14^ and in recent studies of patients with idiopathic pulmonary fibrosis (IPF).^15^,^19^ Given that multiorgan pathologies appear common in ILA subjects, assessment pathways involving a general medicine/dedicated LCS follow-up clinic rather than respiratory services alone warrant consideration.

The high comorbid burden also raises the question of whether, in addition to shared risk factors (age, smoking) between certain comorbidities and ILA development, ILA presence may be a surrogate marker for premature ageing.^20^ Reduced telomere length, a marker of cellular senescence, has been shown to associate with ILAs,^21^ as well as IPF^1622^,^24^ and other fibrosing lung diseases.^25^ There is growing evidence that endothelial dysfunction (likely present in the predominantly vascular comorbid diseases seen in our ILA population) plays a prominent role in IPF pathogenesis,^26^,^29^ and our data suggest this is happening with increased incidence compared with non-ILA subjects, a decade before the development of respiratory symptoms.

FVC has historically been used as a cardinal measure of lung damage when assessing fibrosing lung diseases.^30^ Recent studies studying ILAs from the COPD Gene study have adopted the presence of radiological fibrosis and reduced lung function (FVC<80%, DLCO<70%) as the criteria for suspected ILD.^31 32^ Yet, the high burden of emphysema in a LCS population can result in a relatively diminished FVC value for a given extent of fibrosis.^33^ As a result, spirometric values might appear within the normal range, thereby disguising the presence of fibrosing lung disease.^34^ Our study showed that FVC failed to distinguish ILAs from controls and was limited in identifying subjects with increased mortality risk in a heavy-smoking population. Using CT alone might therefore improve the detection of important ILAs, with the caveat that in subjects with NFILA and FILA, assessment of two time point CTs might be required to ensure CT changes remain persistent.

There were limitations to our study. The COVID-19 pandemic precluded comprehensive follow-up spirometry, limiting evaluation of longitudinal FVC trajectories. We also focused on subjects where ILAs had been identified by the initial reporting radiologist. Given that our ILA prevalence is lower than other LCS studies,^35^ it is probable that some ILAs in SUMMIT were not identified. Accordingly, we are currently examining all the SUMMIT CTs (n=>22 000) to evaluate its true ILA prevalence. Finally, our study examined interobserver variability between just two radiologist pairs. Reproducibility of our scoring system among a larger radiologist population in independent external validation cohorts needs confirmation before it can be considered an alternative to the Fleischner criteria. Further studies should also assess the relevance of our classification to non-smoking populations.

In conclusion, we have defined a reproducible new method to separate clinically important ILAs in LCS populations. We have demonstrated the limitations inherent when using FVC and spirometry in a heavy-smoking study population. Our findings should have relevance when estimating lung damage and comorbidity burden in subjects with ILAs detected as part of LCS.

## Supplementary material

10.1136/bmjresp-2025-003298 online supplemental file 1

## Data Availability

All data relevant to the study are included in the article or uploaded as supplementary information.
