# Supplementary material for: Differentiating clinically important interstitial lung abnormalities in lung cancer screening
Source: BMJ Open Respir Res. 2025 Sep 10;12(1):e003298. doi: 10.1136/bmjresp-2025-003298 (PMC12506105; doi:10.1136/bmjresp-2025-003298)
Supplement: online supplemental file 1 [file bmjresp-12-1-s001.pdf]

**Supplementary**

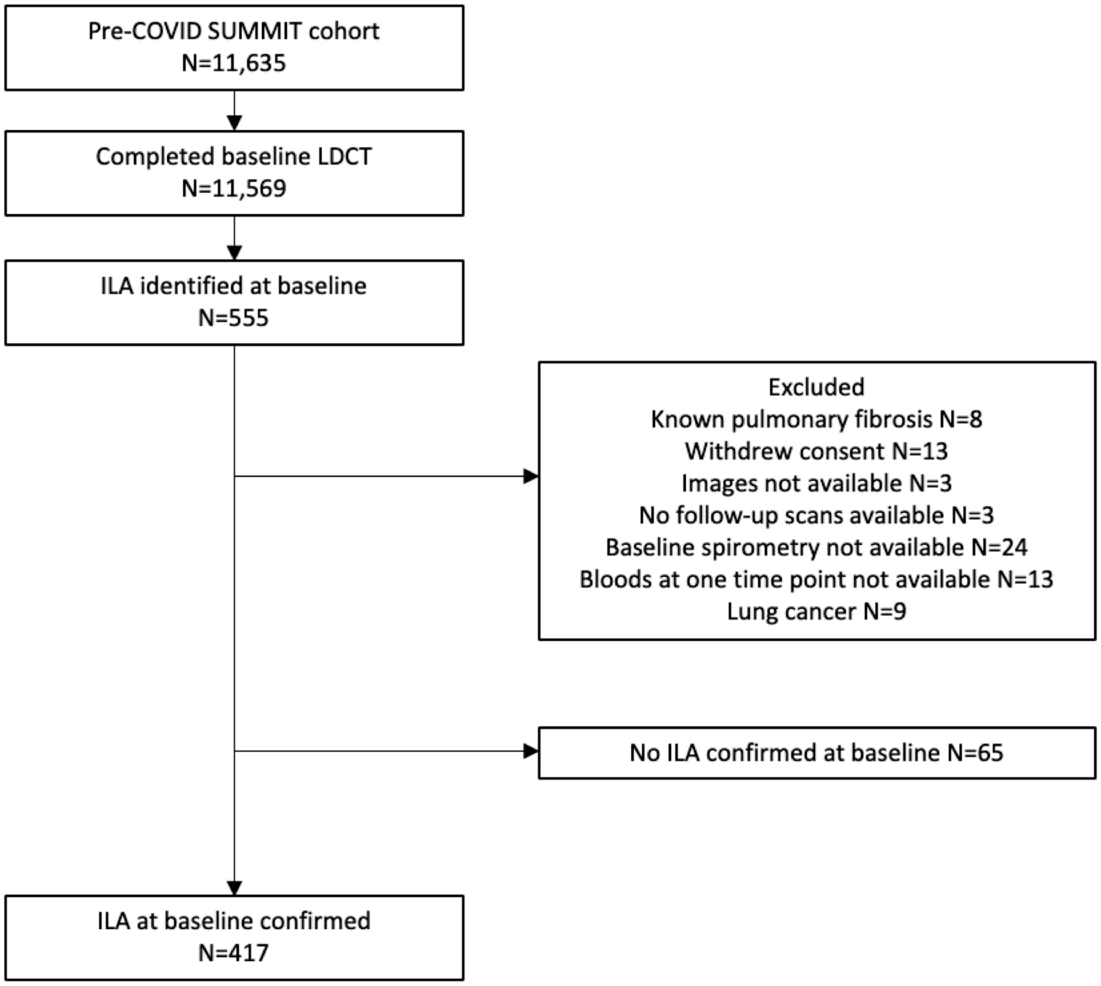


**Supplementary Figure 1.** CONSORT diagram showing the reasons for exclusion of CT scans from SUMMIT study subjects identified as having interstitial lung abnormalities (ILA) on CT.

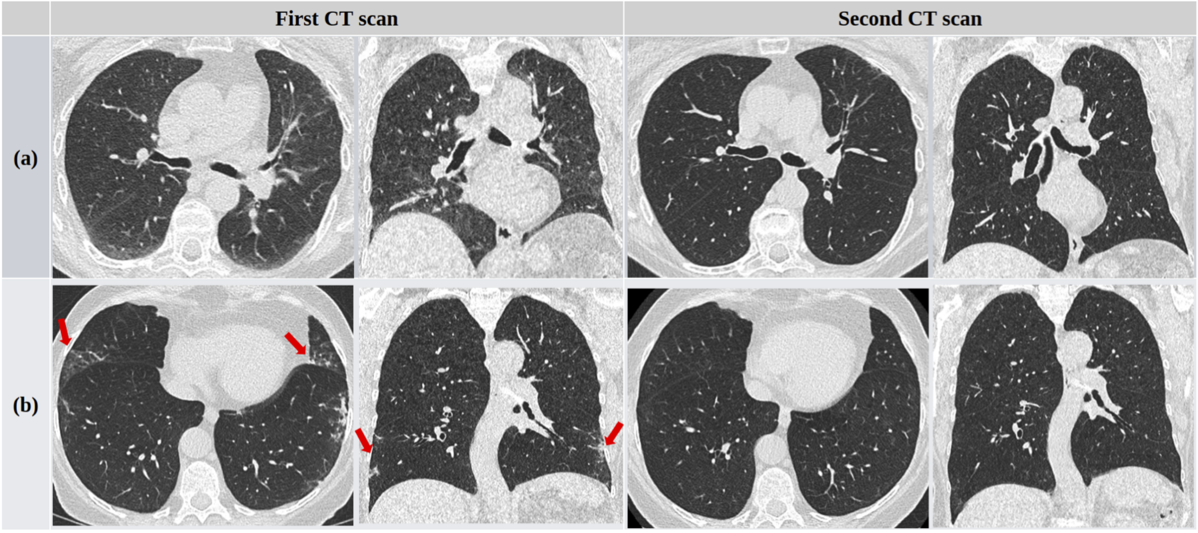


**Supplementary Figure 2.** Two examples of ILA detection by the reporting radiologist which were reclassified as no ILA after review of subsequent timepoint CTs. The first case (top row) shows a poor inspiratory effort on the first CT scan giving the appearance of ground glass infiltrates on the CT. These appearances resolved following acquisition of a good inspiratory CT performed a year later. The second case (bottom row) shows infection on the first CT which was thought to represent ILA by the reporting radiologist. The ground glass and reticular abnormalities (red arrows), resolved on the CT scan performed a year later.

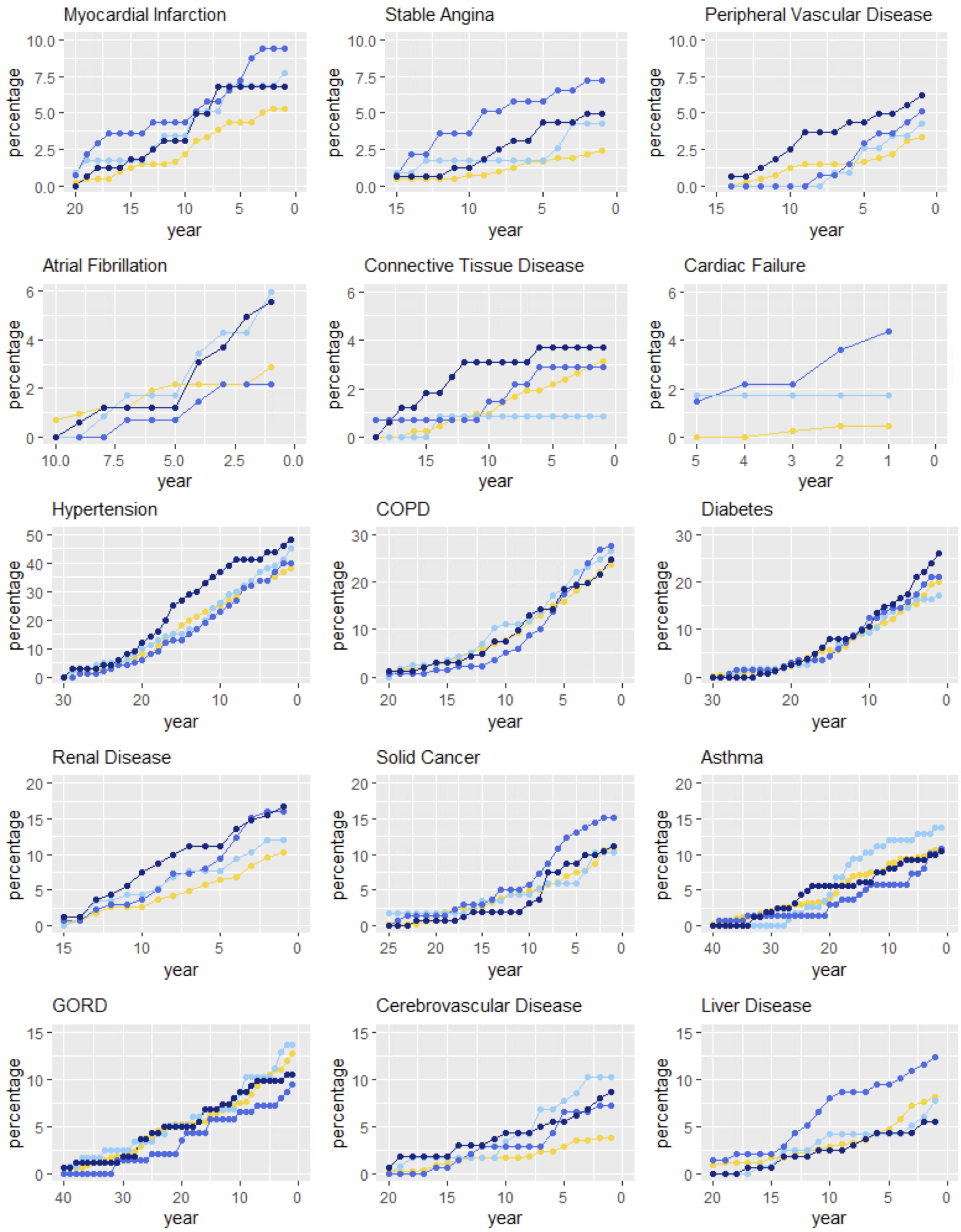


**Supplementary Figure 3**: Curves showing cumulative incidence of disease comorbidities in the years prior to the initial lung cancer screening CT scan. Zero on the x-axis is the date of the initial CT scan. Separate curves are shown for subjects with no interstitial lung abnormalities (ILA), yellow), non-fibrotic ILA (light blue), fibrotic ILA (royal blue) and undiagnosed ILD (UILD)(navy).

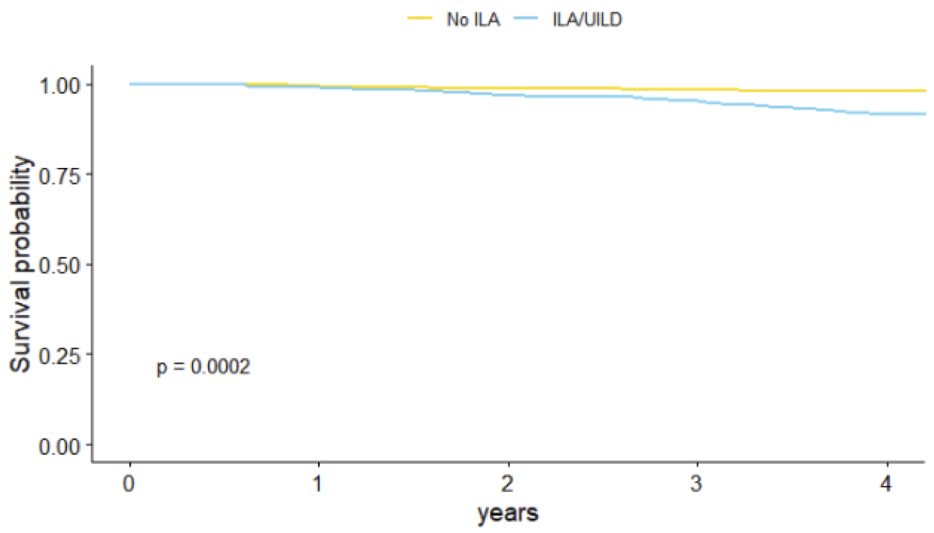


Number at risk

| | | | | | Time |
|---|---|---|---|---|---|
| status | t0 | t1 | t2 | t3 | t4 |
| No ILA | 417 | 415 | 412 | 410 | 408 |
| ILA/UILD | 410 | 406 | 398 | 391 | 378 |

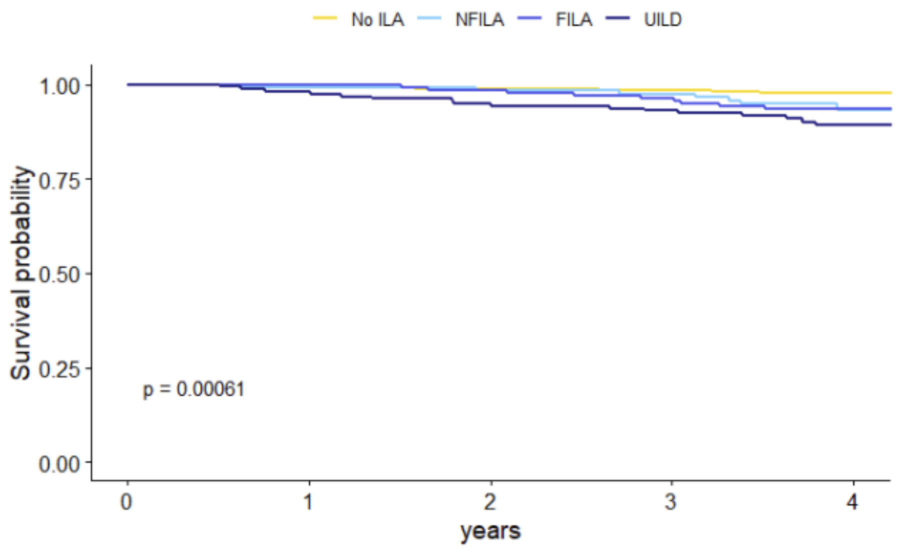


Number at risk

| | | | | | Time |
|---|---|---|---|---|---|
| status | t0 | t1 | t2 | t3 | t4 |
| No ILA | 417 | 415 | 412 | 410 | 408 |
| NFILA | 115 | 114 | 113 | 112 | 108 |
| FILA | 138 | 138 | 136 | 133 | 129 |
| UILD | 157 | 154 | 149 | 146 | 141 |

**Supplementary Figure 4:** Kaplan-Meier Survival Curves for participants with and without interstitial lung abnormalities/disease (ILA) on CT (top); and for participants with and without subtypes of ILA/UILD on CT (bottom). This subanalysis excluded participants who died from COVID-19 infection

| | NFILA | FILA | UILD |
|---|---|---|---|
| Fleischner criteria | 0.42 | 0.26 | 0.51 |
| Proposed criteria | 0.86 | 0.82 | 0.94 |

**Supplementary Table 1:** Comparison of weighted Kappa scores when utilising the Fleischner criteria versus the proposed criteria to detect ILAs and subtypes.

| Disease | 417 ILA/UILD | 417 No ILA | p-value |
|---|---|---|---|
| Hypertension | 187 (45%) | 159 (38%) | 0.058 |
| COPD | 109 (26%) | 99 (24%) | 0.471 |
| Diabetes | 91 (22%) | 83 (20%) | 0.551 |
| Renal Disease | 63 (15%) | 43 (10%) | 0.048* |
| Solid Cancer | 51 (12%) | 45 (11%) | 0.588 |
| Asthma | 48 (12%) | 44 (11%) | 0.740 |
| GORD | 46 (11%) | 53 (13%) | 0.521 |
| Cerebrovascular Disease | 36 (9%) | 16 (4%) | 0.007* |
| Liver Disease | 35 (8%) | 34 (8%) | 1.000 |
| Myocardial Infarction | 33 (8%) | 22 (5%) | 0.163 |
| Stable Angina | 23 (6%) | 10 (2%) | 0.033* |
| Peripheral Vascular Disease | 22 (5%) | 14 (3%) | 0.233 |
| Atrial Fibrillation | 19 (5%) | 12 (3%) | 0.272 |
| Connective Tissue Disease | 11 (3%) | 13 (3%) | 0.836 |
| Cardiac Failure | 8 (2%) | 2 (<1%) | 0.112 |
| Osteoporosis | 8 (2%) | 9 (2%) | 1.000 |
| Blood Cancer | 5 (1%) | 6 (1%) | 1.000 |
| Bronchiectasis | 2 (<1%) | 2 (<1%) | 1.000 |

**Supplementary Table 2:** Frequency of prevalent comorbidities at enrolment in the SUMMIT study in subjects with and without interstitial lung abnormalities. The GORD (Gastro-oesophageal reflux disease) category included peptic ulcer disease. Blood cancer included leukaemia and lymphoma. Solid cancers represented cancer with metastases, metastatic solid tumours and solid organ malignancies. Cardiac failure included cor pulmonale and congestive heart failure. COPD=chronic obstructive pulmonary disease; ILA = interstitial lung abnormality, UILD = undiagnosed interstitial lung disease.

| **Disease** | **117 NFILA** | **138 FILA** | **162 UILD** |
|---|---|---|---|
| Hypertension | 53 (45%) | 56 (41%) | 78 (48%) |
| COPD | 31 (26%) | 38 (28%) | 40 (25%) |
| Diabetes | 20 (17%) | 29 (21%) | 42 (26%) |
| Renal Disease | 14 (12%) | 22 (16%) | 27 (17%) |
| Solid Cancer | 12 (10%) | 21 (15%) | 18 (11%) |
| Asthma | 16 (14%) | 15 (11%) | 17 (10%) |
| GORD | 16 (14%) | 13 (9%) | 17 (10%) |
| Cerebrovascular Disease | 12 (10%) | 10 (7%) | 14 (9%) |
| Liver Disease | 9 (8%) | 17 (12%) | 9 (6%) |
| Myocardial Infarction | 9 (8%) | 13 (9%) | 11 (7%) |
| Stable Angina | 5 (4%) | 10 (7%) | 8 (5%) |
| Peripheral Vascular Disease | 5 (4%) | 7 (5%) | 10 (6%) |
| Atrial Fibrillation | 7 (6%) | 3 (2%) | 9 (6%) |
| Connective Tissue Disease | 1 (1%) | 4 (3%) | 6 (4%) |
| Cardiac Failure | 2 (2%) | 6 (4%) | 0 |
| Osteoporosis | 2 (2%) | 1 (1%) | 5 (3%) |
| Blood Cancer | 3 (3%) | 2 (1%) | 0 |
| Bronchiectasis | 0 | 0 | 2 (1%) |

**Supplementary Table 3:** Frequency of prevalent comorbidities at enrolment in the SUMMIT study in interstitial lung abnormality subgroups. The GORD (Gastro-oesophageal reflux disease) category included peptic ulcer disease. Blood cancer included leukaemia and lymphoma. Solid cancers represented cancer with metastases, metastatic solid tumours and solid organ malignancies. Cardiac failure included cor pulmonale and congestive heart failure. COPD=chronic obstructive pulmonary disease; NFILA=non-fibrotic interstitial lung abnormality FILA=fibrotic interstitial lung abnormality, UILD= undiagnosed interstitial lung disease.

| | No ILA | | ILA/UILD | | NFILA | | FILA | | UILD | |
|---|---|---|---|---|---|---|---|---|---|---|
| Time | Y0 | Y2 | Y0 | Y2 | Y0 | Y2 | Y0 | Y2 | Y0 | Y2 |
| N | N=366 | | N=326 | | N=94 | | N=115 | | N=118 | |
| No. with cough (%) | 126 (34.4) | 89 (24.3) | 115 (35.3) | 71 (21.8) | 36 (38.3) | 20 (21.3) | 42 (36.5) | 28 (24.4) | 37 (31.4) | 23 (19.5) |
| No. with follow up mMRC score | N=362 | | N=320 | | N=94 | | N=113 | | N=113 | |
| Average mMRC score | 0.89 | 0.82 | 0.97 | 1.02 | 0.89 | 0.98 | 1.04 | 1.05 | 0.96 | 1.01 |
| No. with mMRC ≥ 2(%) | 63 (17.4) | 64 (17.7) | 65 (20.3) | 79 (24.7) | 19 (20.2) | 24 (25.5) | 24 (21.2) | 30 (26.5) | 22 (19.5) | 25 (22.1) |
| No. with worsening mMRC score (%) | | 68 (18.8) | | 86 (26.9) | | 24 (25.5) | | 28 (24.8) | | 34 (30.1) |

**Supplementary Table 4:** Symptom reports of cough and modified Medical Research Council (mMRC) breathlessness scores at enrolment in the SUMMIT study (Y0) and on questionnaires performed two years later (Y2). Results are shown for subjects with and without interstitial lung abnormalities (ILA), and for ILA subgroups: NFILA=non-fibrotic interstitial lung abnormality, FILA=fibrotic interstitial lung abnormality, UILD=undiagnosed interstitial lung disease.

| Covariate | HR | LHR | UHR | P-value |
|---|---|---|---|---|
| FVC | 0.98 | 0.96 | 1.00 | 0.04 |
| Age | 1.11 | 1.05 | 1.18 | <0.001 |
| Smoking pack years | 1.02 | 1.01 | 1.03 | <0.001 |
| Gender (Male) | 1.83 | 0.88 | 3.82 | 0.11 |
| ILA/UILD Presence | **4.90** | 2.36 | 10.10 | **<0.001** |

**Supplementary Table 5**: Multivariable Cox regression analyses for mortality comparing the newly proposed criteria classification of UILD/FILA/NFILA v no ILA based on traction bronchiolectasis (n=834). NFILA=non-fibrotic interstitial lung abnormality FILA=fibrotic interstitial lung abnormality, UILD=undiagnosed interstitial lung disease.

| Covariate | HR | LHR | UHR | P-value |
|---|---|---|---|---|
| FVC | 0.96 | 0.94 | 0.99 | 0.002 |
| Age | 1.11 | 1.03 | 1.20 | 0.007 |
| Smoking pack years | 1.02 | 1.00 | 1.03 | 0.04 |
| Gender (Male) | 1.89 | 0.71 | 5.01 | 0.20 |
| NFILA/FILA presence | 4.45 | 1.94 | 10.2 | **<0.001** |

**Supplementary Table 6:** Multivariable Cox regression analyses for mortality comparing the newly proposed classification of FILA/NFILA (undiagnosed interstitial lung disease removed) v no ILA (n=672). NFILA=non-fibrotic interstitial lung abnormality FILA=fibrotic interstitial lung abnormality.

| Covariate | HR | LHR | UHR | P-value |
|---|---|---|---|---|
| FVC | 0.98 | 0.96 | 1.00 | 0.02 |
| Age | 1.11 | 1.05 | 1.18 | 5.33E-04 |
| Smoking pack years | 1.15 | 0.64 | 2.07 | 0.64 |
| Gender (Male) | 1.69 | 0.81 | 3.55 | 0.16 |
| ILA/UILD Presence | **3.92** | 2.09 | 7.34 | **2.00E-05** |

**Supplementary Table 7**: Multivariable Cox regression analyses for mortality comparing the Fleischner Criteria classification of UILD/FILA/NFILA v no ILA (n=834). NFILA=non-fibrotic interstitial lung abnormality FILA=fibrotic interstitial lung abnormality, UILD=undiagnosed interstitial lung disease.

| Covariate | HR | LHR | UHR | P-value |
|---|---|---|---|---|
| FVC | 0.99 | 0.97 | 1.01 | 0.22 |
| Age | 1.12 | 1.04 | 1.20 | 2.08E-03 |
| Smoking_ pack years | 1.39 | 0.69 | 2.79 | 0.36 |
| Gender (Male) | 1.72 | 0.74 | 4.00 | 0.21 |
| NFILA/FILA presence | **3.31** | 1.64 | 6.67 | **8.12E-04** |

**Supplementary Table 8 :** Multivariable Cox regression analyses for mortality comparing the Fleischner criteria classification of FILA/NFILA (undiagnosed interstitial lung disease removed) v no ILA (n=723). NFILA=non-fibrotic interstitial lung abnormality FILA=fibrotic interstitial lung abnormality.

| Number of participants (No. of deaths) | No ILA | NFILA | FILA | UILD |
|---|---|---|---|---|
| Proposed Criteria | 417(9) | 117(9) | 138(9) | 162(21) |
| Fleischner | 512(14) | 181(18) | 30(1) | 111(15) |

**Supplementary Table 9:** Comparison of ILA group allocations and deaths using the proposed criteria versus Fleischner criteria. NFILA=non-fibrotic interstitial lung abnormality FILA=fibrotic interstitial lung abnormality, UILD=undiagnosed interstitial lung disease.

**Appendix 1**

**Cancer risk criteria for inclusion into the SUMMIT study included:** meeting the USPSTF (2013) criteria [37] or a Prostate, Lung, Colorectal and Ovarian 2012 model ($PLCO_{M2012}$) 6-year cancer risk threshold of ≥1.3% [38], and required that participants were not currently receiving treatments for an active cancer (except adjuvant hormonal therapy).
